# Multiplex gene precise editing and large DNA fragment deletion by the CRISPR‐Cas9‐TRAMA system in edible mushroom Cordyceps militaris

**DOI:** 10.1111/1751-7915.14147

**Published:** 2022-09-22

**Authors:** Bai‐Xiong Chen, Ling‐Na Xue, Tao Wei, Na Wang, Jing‐Ru Zhong, Zhi‐Wei Ye, Li‐Qiong Guo, Jun‐Fang Lin

**Affiliations:** ^1^ Institute of Food Biotechnology & College of Food Science South China Agricultural University Guangzhou China; ^2^ Research Center for Micro‐Ecological Agent Engineering and Technology of Guangdong Province Guangzhou China; ^3^ Guangzhou Alchemy Biotechnology Co., Ltd Guangzhou China

## Abstract

The medicinal mushroom Cordyceps militaris contains abundant valuable bioactive ingredients that have attracted a great deal of attention in the pharmaceutical and cosmetic industries. However, the development of this valuable mushroom faces the obstacle of lacking powerful genomic engineering tools. Here, by excavating the endogenous tRNA‐processed element, introducing the extrachromosomal plasmid and alongside with homologous template, we develop a marker‐free CRISPR‐Cas9‐TRAMA genomic editing system to achieve the multiplex gene precise editing and large synthetic cluster deletion in *C. militaris*. We further operated editing in the synthetases of cordycepin and ergothioneine to demonstrate the application of Cas9‐TRAMA system in protein modification, promoter strength evaluation and 10 kb metabolic synthetic cluster deletion. The Cas9‐TRAMA system provides a scalable method for excavating the valuable metabolic resource of medicinal mushrooms and constructing a mystical cellular pathway to elucidate the complex cell behaviours of the edible mushroom.

## Graphical abstract

This study identified the endogenic tRNA processed element of mushroom crop *Cordyceps militaris*, and proved the *in vivo* generation of gRNA by promoter P*trpC* could highly improve the editing efficiency of genomic editing. By introducing the extra‐chromosomal plasmid and alongside with homologous template, a marker‐free CRISPR‐Cas9‐TRAMA genomic editing system was built to achieve the multiplex gene precise editing and large synthetic cluster deletion in *C. militaris*.

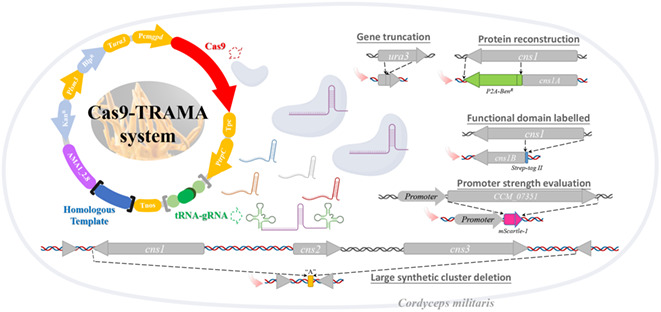

## INTRODUCTION

*Cordyceps militaris* is a well‐known medicinal mushroom in East Asia for centuries (Cui, 2015). It contains lots of valuable bioactive compounds (Chen et al., 2021) such as cordycepin, *Cordyceps* polysaccharide, carotenoid and ergothioneine. As the applications of high‐throughput sequencing analysis (Chen et al., 2020; Zheng et al., 2011) and genome editing technologies (Chen et al., 2018) in *Cordyceps*, *Cordyceps militaris* have attracted much attention in the pharmaceutical and cosmetic industries. For example, *C. militaris* has been engineered as an industrial chassis cell to convert spent mushroom substrate into anticancer drug pentostatin recently (Zou, Li, et al., 2021). However, frequent degeneration (Lou et al., 2019) with lower ingredient production in serial subculturing and preservation are the main obstacles to strict the *C. militaris* industrial application.

The first genetic modified (preventing from turning brown) mushroom *A. bisporus* received regulatory declaration by the US Department of Agriculture (USDA) since 2016 (Waltz, 2016; Wesseler et al., 2022), which implied the great potential of gene‐edited mushroom application in global food or nutritive fortifier markets. The CRISPR/Cas9 genome editing technology has also been applied to other mushroom, containing *Pleurotus ostreatus* (Boontawon, Nakazawa, Horii, et al., 2021; Boontawon, Nakazawa, Inoue, et al., 2021; Boontawon, Takehito, Xu, et al., 2021), *P. eryngii* (Wang et al., 2021), *Ganoderma lucidum* (Liu et al., 2020; Qin et al., 2017; Tu et al., 2021; Wang et al., 2020), *Schizophyllum commune* (Jan Vonk et al., 2019), *Coprinopsis cinerea* (Sugano et al., 2017), *Lentinula edodes* (Moon et al., 2021) and *C. militaris* (Chen et al., 2018; Zou, Xiao, et al., 2021). But some of these applications were reported to function only in specific genes, which process morphological or grown characteristic change function such as *ura3*/*pyrG* (the orotidine‐5′‐phosphate decarboxylase gene), which endow the mutant could be easily distinguishable from abundant transformants without performing sequencing. Also, most of them are facing the problems of low editing efficiency, disable multiplex target editing and larger cluster deletion.

Because of the two‐stage ontogenetic processing, the mushroom has a much more complex genetic background than micro‐fungi. Except for the limited selection of markers and molecular tools, the main technical bottleneck in improving the editing efficiency of the CRISPR‐Cas9 system in mushroom was the maintenance of abundant mature gRNA in a short period. Considering the easily degradable characteristic of small molecular RNA, the gRNA was designed to be continually synthesized by in vivo gRNA cassette or directly transformed from in vitro (Tang et al., 2019), including the strategy of driving with the RNA polymerase III (Pol III) promoter, introducing exogenous Hammerhead (HH) and hepatitis delta virus (HDV) ribozymes, fusing with Csy4 RNA ribonuclease (Čermák et al., 2017) and applying endogenous tRNA‐processing system.

In this study, we excavated the endogenic tRNA modification element to perform in vivo RNA processing. Subsequently leveraging the multiplex RNA‐processing function of tRNA element, we optimized the previous *C. militaris* CRISPR‐Cas9 gene deletion system to build a one‐step multiplex gene editing system in a single transcript unit. When further coupled with the modified autonomously replicating AMA1 (Autonomously Maintained in *Aspergillus)* element (Sarkari et al., 2017), together with the homology template of target sites, we built the Cas9‐TRAMA genomic editing system, which processed a powerful non‐selective editing function for intact coding gene to perform replacement, deletion and modification via NHEJ (non‐homologous end‐joining) or HDR (homology‐directed repair). Most importantly, this system could easily be removed after performing the genomic editing. In addition, to demonstrate that the Cas9‐TRAMA system enables a wider range of genome engineering application, we operated genomic editing on the synthetases of cordycepin and ergothioneine and successfully achieved protein modification, promoter strength evaluation and 10 kb metabolic synthetic cluster deletion in *C. militaris*. The Cas9‐TRAMA editing system provides a scalable method to excavate the valuable metabolic resource of edible mushrooms and to engineer a mystical cellular pathway to elucidate the complex cell behaviours of mushroom‐forming fungi.

## EXPERIMENTAL PROCEDURES

### Strains, plasmids and growth conditions

*Escherichia coli* DH5α and *Agrobacterium tumefaciens* AGL‐1 (Weidi Bio, Shanghai, China) were used for vector construction and fungal transformation, respectively. *C. militaris* CM15 was wild type of farming strain and used as the host for gene editing. *C. militaris* was grown on potato peptone dextrose agar (PDA) or M‐100 medium (H_3_BO_3_ 30 μg/L, MnCl_2_·4H_2_O 70 μg/L, ZnCl_2_ 200 μg/L, Na_2_MoO_4_·2H_2_O 20 μg/L, FeCl_3_·6H_2_O 50 μg/L, CuSO_4_·5H_2_O 200 μg/L, KH_2_PO_4_ 1 g/L, Na_2_SO_4_ 0.25 g/L, KCl 0.5 g/L, MgSO_4_·7H_2_O 125 mg/L, CaCl_2_ 62.5 g/L, Glucose 10 g/L, KNO_3_ 3 g/L, agar 20 g/L) at 25°C in dark. The backbone vector p390‐blpR‐cmcas9‐gfp was constructed in our previous study (Chen et al., 2018).

### Vector construction

The synthetic DNA sequence, plasmids and primers used for vector construction in this study were listed in the Tables S1–S3. The constructed methods (Table S2) were performed as the suggestion of standard protocols from the company or modified based on the theory reported in ADDGENE (http://www.addgene.org/), including Hypha‐direct PCR (KOD FX, TOYOBO CO., LTD., Japan), OE‐PCR, Restriction enzyme ligation (FastDigest Restrict Enzyme, Thermo Fisher Scientific, USA; Ligation high Ver.2, TOYOBO Co., Ltd., Japan), BioBrick assembly, primers annealing adapter linker, Gibson assembly and Golden gate cloning. The multiple tRNA‐gRNA elements were assembled as described in Figure S4.

### sgRNA design and transformation in *C. militaris*

The design of sgRNA and *C. militaris* transformation via ATMT method were performed in our previous study (Chen et al., 2018). The PEG‐mediated protoplast transformation was also performed as previously but with a slight modification. The generation of protoplasts was carried out in an STC buffer with 0.006% Triton X‐100 and incubated in M‐100 medium with glufosinate ammonium. The target site sequencing of transformants was performed by GENEWIZ (Suzhou, China) or Tsingke Biotechnology (Beijing, China).

### tRNA prediction of *C. militaris*

The genomic DNA sequence of C. militaris was downloaded from NCBI (https://www.ncbi.nlm.nih.gov/) with accession number GCA_000225605. The detection and classification of tRNA genes were performed using the online tool tRNAscan‐SE 2.0 (Chan et al., 2021) with the default setting. The output tRNA sequence was subsequently uploaded to the online tool MAFFT (FFT‐NS‐I, version 7.397) (Katoh & Standley, 2013) to perform multiple sequence alignment. Besides, the picked tRNA element was uploaded to perform RNA structure prediction by software RNA structure (Reuter & Mathews, 2010) with a default setting.

## RESULTS AND DISCUSSION

### Identification of tRNA‐processed element for single transcript unit CRISPR‐Cas9 system for ura3 editing

A CRISPR‐Cas9 gene disruption system in *C. militaris* was constructed in our previous study (Chen et al., 2018), but its application faces several obstacles. We have demonstrated a single transcript unit processing strategy of gRNA based on HH and HDV ribozymes, which was successfully used in many other species but did not function in *C. militaris*. Therefore, we optimized the gRNA transcript unit by excavating the endogenous tRNA‐processed system rather than exogenous ribozymes, as known that pre‐tRNA contains a conserved sequence that will be processed by RNase Z (Canino et al., 2009)/P (Gutmann et al., 2012). So, we first excavated the tRNA sequence of *C. militaris* with sequence alignment and total obtained 118 tRNAs (Figure S1a, Table S4). Among them, 7 tRNA^Gly^ sequences showed a highly similar conserved sequence (Figure S1b). We selected 87 bp as the element to perform RNA‐processing as the length of tRNA used in rice plant (Xie et al., 2015).

**FIGURE 1:**
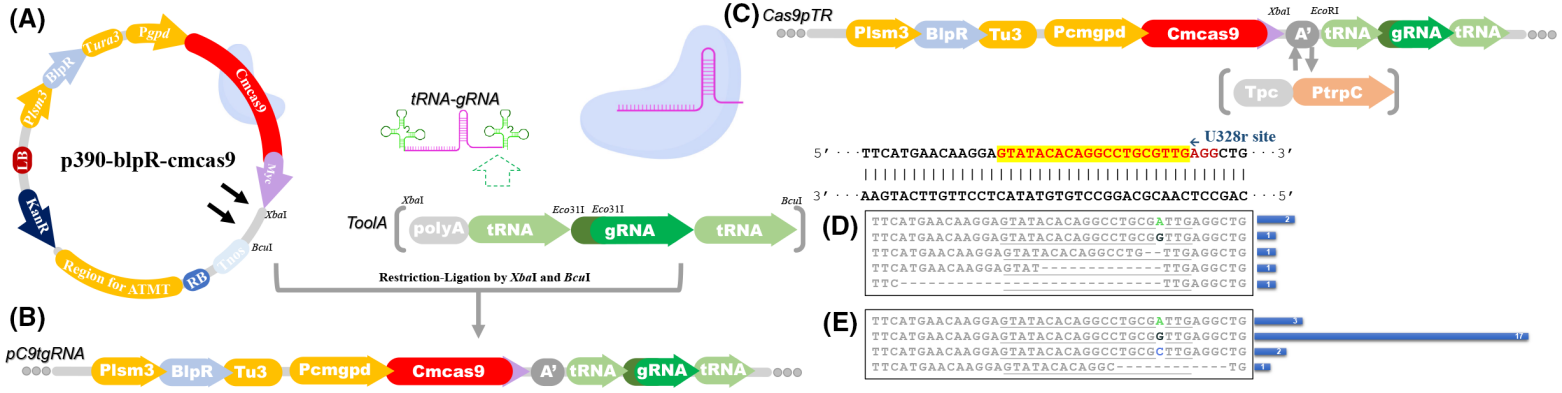
The diagram of vector construction (A) of pC9tgRNA (B) and Cas9pTR (C) for the single transcript unit CRISPR‐Cas9 TR system. Partly sequence (5′–3′) of the target site U328r in mutants (the blue bar represented the number of mutants with the corresponding mutation) generated by pC9tgRNA (D) or Cas9pTR (E).

To apply the precise processing ability of tRNA genes to produce small RNAs, we inserted the gRNA fragment, along with the conserved tRNA genes on both sides, into the Cas9‐expressed vector (Chen et al., 2018). Since few strong promoters have been reported to be used in *C. militaris*, the gRNA cassette shared the promoter of Cas9 in the primary version. This tRNA‐gRNA‐tRNA fragment was fused to the downstream of Cas9 (Figure 1A) to construct a single transcript unit CRISPR‐Cas9 system (the vector was named pC9tgRNA‐U328r, Figure 1B) in *C. militaris*. Through single transformation via ATMT (Agrobacterium tumefaciens‐mediated transformation) method, six mutons were obtained from cultivated plate with selective pressure of 5‐FOA (5‐fluoroorotic acid). The *ura3* gene of these mutons occurred by single base insertion and 2‐28 bp deletion at 3 bp upstream of the target's PAM sites (Figure 1D, Table S5), which consisted of the editing pattern of our previous Cas9 editing study. With the selective pressure in the medium as previously, the number of positive mutons was as same as the previous editing method with the transformation of presynthesized gRNA. It indicated that this tRNA^Gly^ element could process the gRNA sequence to be functional in single unit CRISPR‐Cas9 genomic editing.

Although the editing was successful in *ura3*, the rather low editing efficiency implied that this system was not suitable for editing genomic sequences without proper selective pressure. We suspected that the main problem was the transcript level of gRNA sequence. The quantity of mature gRNA processed from giant *cas9‐tRNA‐gRNA‐tRNA* mRNA unit was too low to effectively assist the target recognition of cas9. We thereby inserted a terminator to separate their reading frame and added a medium strength promoter P*trpC* to drive the tRNA‐gRNA fragment. This vector was named as Cas9pTR‐U328r (Figure 1C) and then transformed into *C. militaris* with ATMT. As a result (Figure 1E, Table S5), the editing efficiency of this Cas9‐TR system increased nearly 4 times over that of pC9tgRNA‐U328r. There were four types of mutations occurring in 23 *ura3* mutons, containing a 12 bp deletion and three 1 bp insertion, while 17 were generated insertion with ‘G' in 3 bp upstream of the PAM sites. It indicated that the combination of P*trpC* promoter and tRNA element could be efficient to drive the transcription of activating gRNA, and the Cas9‐TR system is capable for editing genes of connecting to auxotroph or resistance in *C. militaris*.

### Genomic sequence truncation with double site editing by the CRISPR Cas9‐TR system

**FIGURE 2:**
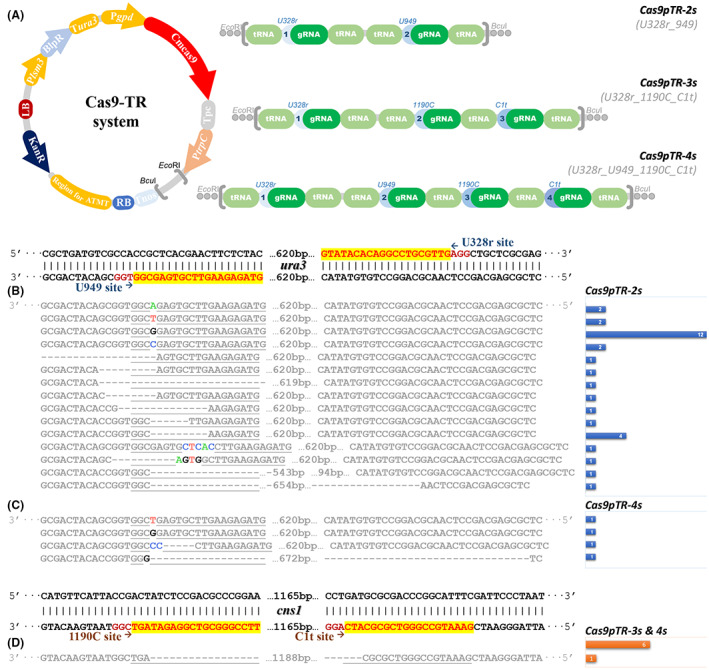
The diagram of constitution of vector Cas9pTR vectors (A). The DNA sequence and number of mutons generated by the Cas9‐TRs system, which contained the mutons, showed the *ura3* mutation with Cas9pTR‐2s (B) and Cas9pTR‐4s (C), and the mutons showed the *cns1* mutation with Cas9pTR‐3s and Cas9pTR‐4s (D).

The array of tRNA‐processed elements is capable of modifying multiple small RNA in a single cassette. To test the multiple processing function of the tRNA^Gly^ element, we constructed a tandem that manipulated the generation of two or more gRNAs as shown in Figure 2A. The double editing was targeting the U328r and U949 sites of *ura3*. After transformation with vector Cas9pTR‐2s and selection by 5‐FOA, we obtained 35 *ura3* mutations (Figure 2B, Table S5), containing 19 insertions, 1 replacement and 15 deletions. All the mutons generated mutation in site U949, except for one simultaneously showed deletion in U328r site, which was resulting in a 654 bp sequence truncation of *ura3*. As in the last section, the insertion of ‘G’ in 3 bp upstream of the PAM site obtained individuals more than others. The deletions were generated with the random pattern, but all the mutons lost four bases upstream of the PAM site. Also, a 543 bp sequence was strangely deleted from 3 bp upstream of U949 site to 114 bp upstream of the U328r site. Our previous study indicated that the U328r site was a valid target for editing by the CRISPR/Cas9 system (Figure 1D,E), but the results in Figure 2B,C showed that all the cutting sites were located at U949 while the cutting of U328r was only performed in double sites truncation. It implied that the first sgRNA generated by the double tRNA‐gRNA‐tRNA tandem will suffer from low‐efficiency recognition of Cas9. And once the first site was successfully edited, the double site truncation will supervenient generate.

In addition, we constructed vectors with three or four target sites. These new sites targeted at regular bio‐synthetase genes to validate the general applicability of the Cas9‐TRs system. During the selection of transformants, the medium did not contain additives that generated selective pressure on target genes to test the multiplex sequence edited efficiency of the Cas9‐TRs system. The vector Cas9pTR‐3s (Figure 2A), which contains three target sites, was transformed using the ATMT method and cultivated on a medium containing glufosinate ammonium. We randomly picked 40 transformants and obtained 6 mutons with edited in site 1190C and C1t, they showed 1188 bp sequence truncation from 3 bp upstream of the PAM site in 1190C and C1t, respectively (Figure 2D, Table S5). Unfortunately, no *ura3* mutation was obtained within these 40 transformants. While performing transformation with vector contained 4 gRNA target sites (Figure 2A) and they were uniformly distributed in two genes (*ura3* and *cns1*), we only obtained one *cns1* truncated muton (Figure 2D, Table S5) and four *ura3* edited mutons (Figure 2C, Table S5) from 40 randomly selected transformants. None of them showed three or four sites edited simultaneously. It suggested that the Cas9‐TRs system is capable for double site truncation in regular genes but not for the single, triple or more sites editing if the corresponding selective pressure is absent. Most edited mutons occurred mutation with single base insertion or several bases deletion in 3 bp upstream of the PAM site, while mutons with single site editing and fragment replacement in most genes were hard to obtain. Nevertheless, though the editing efficiency was rather low while duelling with genes without selective pressure, the Cas9‐TRs system is capable of deletion via two closed gRNA recognized sites, which implied that it could be used for the verification of functions of most genes via ATMT transformation in C. militaris.

### Construct an efficient multiplex editing system Cas9‐TRAMA with marker‐free vector in *C. militaris*

**FIGURE 3:**
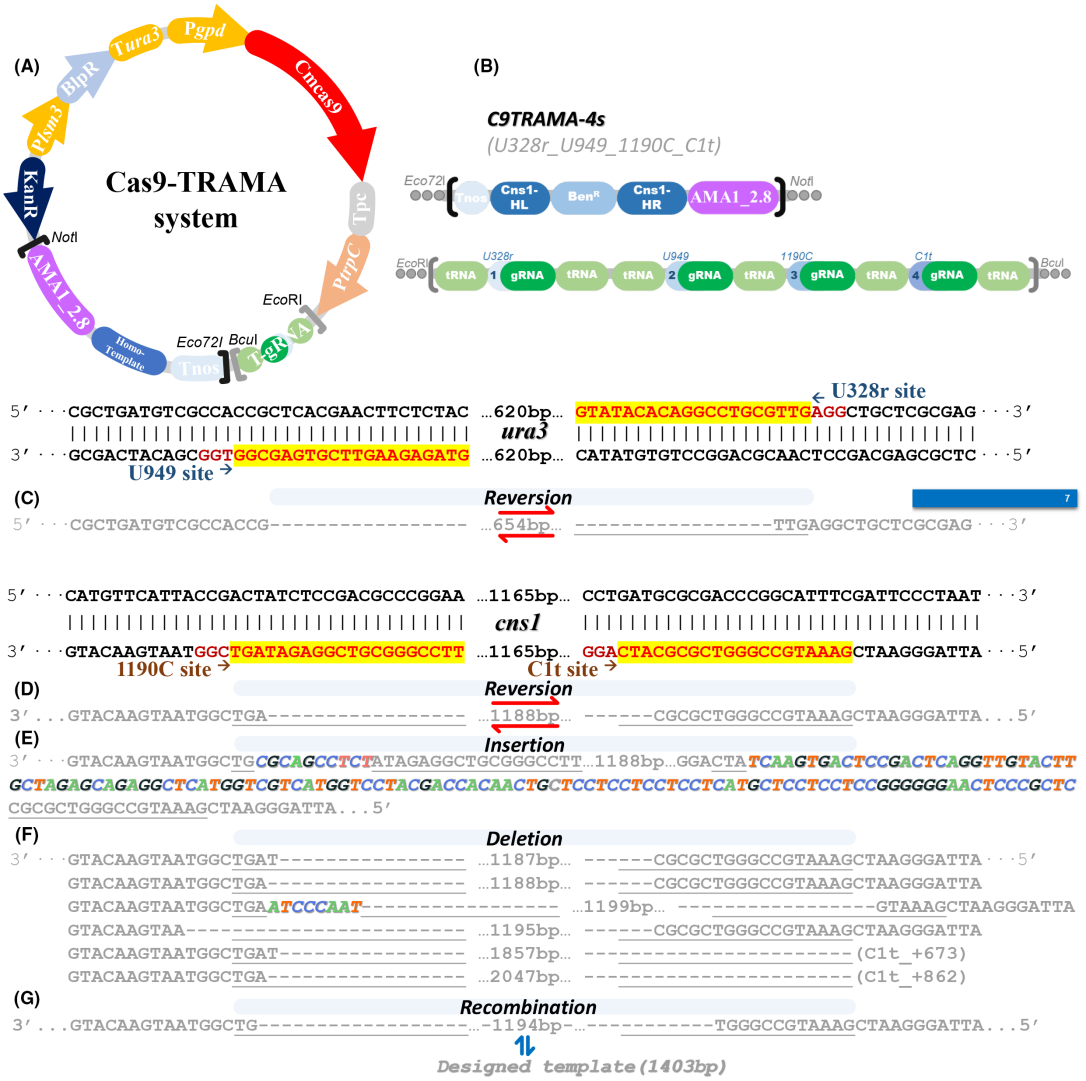
The diagram of constitution of the Cas9‐TRAMA system (A) and constitution of vector C9TRAMA‐4s (B). The DNA sequence and number of *ura3* mutons generated by C9TRAMA‐4s (C). Partly DNA sequence of *cns1* mutons generated by C9TRAMA‐4s contained mutation with reversion (D), insertion (E), deletion (F) and recombination (G).

The previous section indicated the Cas9‐TRs system faced problems with efficient editing sites in *C. militaris* genome. The Cas9‐TR system was performed with open reading fragments inserted into the genome, so theoretically, gRNA and Cas9 will continually generate and perform target sequence cutting until the target sequence was broken. In fact, we obtained many transformants carrying the intact fragment of the TR system, but no mutation of the target site was detected in these transformants. Based on the data of the transcriptome we presented before (Chen et al., 2020), the mature strain of *C. militaris* possesses a strong Synthesis‐Dependent Strand Annealing of Homologous Recombination (SDSA‐HR) pathway, we speculated that this pathway may be functional in fixing DSB caused by Cas9‐gRNA, so most mutations were generated only in the germinated stage and the editing ability to continuously expressing Cas9 was restricted. However, the amount of Cas9‐gRNA generated from the Cas9‐TR system reached a sufficient level and was delayed than the genomic replicated period. To increase the amount of Cas9 and gRNA, we have tried to construct a two‐step gene disruption system, consisting of a Cas9‐overexpressed *C. militaris* strain and in vitro synthesis of mature gRNA (Chen et al., 2018). But the two‐step editing system could only work for the *ura3* gene. Limited by usable molecular elements, to further promote the generation of Cas9‐gRNA, the copies of their synthesized clusters must be increased. Therefore, we introduced the AMA1 element, which was used in *Aspergillus sp*. and *Penicillium chrysogenum* to construct extrachromosomal plasmid (Ouedraogo & Tsang, 2020), to apply in the *C. militaris* CRISPR Cas9 system. Using the *Agrobacterium* vector of the TR system as a backbone, the functional region used in *Agrobacterium* was replaced by the AMA1_2.8 DNA sequence (Sarkari et al., 2017), which was a short version that eliminated the half‐inverted repeat of regular AMA1 element. The AMA1_2.8 fragment was a mitotically unstable and autonomously replicating element. This fragment could endow the plasmid to process the characteristic of ready‐to‐lose in the stage of propagation without selective pressure (Sarkari et al., 2017); it means that the vector could be easily removed and performs a minimum toxicity from the functional editing cluster. It also suggested that the AMA1_2.8 vector could carry the homology template for target sites to endow the editing system capable of editing targets via homologous‐directed recombination. So, we constructed a vector C9TRAMA and subsequently transformed it into *C. militaris* via PEG‐mediated protoplast transformation to build the Cas9‐TRAMA system (Figure 3A).

The ATMT function region of the Cas9pTR‐4s vector was replaced to AMA1_2.8 element, and together with the insertion of *cns1* homologous template, the Cas9‐TRAMA‐4s vector (Figure 3B) was constructed to value the efficiency of this optimized editing. After transforming the 1 μg vector into *C. militaris* via the PEG method and cultivating on the glufosinate ammonium plate to select the individuals that carry the TRAMA vector, we obtained 39 mutons with double site editing under the cultivated condition without selective pressure of targets. These mutons contained 21 individuals who showed double site editing in 1190C/C1t and resulted in a deletion of the 1187‐bp length sequence of *cns1* (Figure 3F, Table S5). Among them, 7 mutons performed simultaneously a 654 bp length sequence reversion in *ura3* between 3 bp upstream of PAM sites of target U949 and U328r (Figure 3C, Table S5). 17.9% (7/39) of mutons with four simultaneous editing sites suggested that the Cas9‐TRAMA system is capable of efficiently editing multiple sites in the genome via the NHEJ pathway of *C. militaris*.

Besides, four mutons showed sequence reversion between 3 bp upstream of PAM sites of 1190C and C1t (Figure 3D, Table S5); another four showed double insertions with 11 bp in 1190C and 119 bp in C1t (Figure 3E, Table S5). And 10 mutons performed homology recombination as designed for the *cns1* gene, which showed an original 1165 bp sequence replaced by a 1403 bp coding gene (Figure 3G, Table S5). Unexpectedly, 25.6% of mutons (10/39) successfully achieved the coding gene reconstruction by the Cas9‐TRAMA system via the HDR pathway of *C. militaris*. Due to the low efficiency of the former genomic editing (Lou et al., 2018) based on homologous recombination, the HDR pathway in most mushroom was regarded to be too weak to assist in genome editing. It suggested that the low efficiency of the former genomic editing in mushroom may not be blamed on the repairing pathway.

The purpose of raising the editing efficiency by increasing the copies of the synthetic cluster has proved to work. Therefore, we tried to further improve recombined efficiency with the TRAMA system by increasing the plasmid amount and/or supplementing the extra liner homology template DNA during the PEG transformation. The results (Figure 3) showed that the editing efficiency was indeed improved with the double amount of plasmid, we total obtained 36 mutons with recombination, 11 mutons with fragment truncation and one fragment reversion. However, the transformation with the combination of 2 μg plasmid and 3 μg template DNA could only generate 9 *cns1* mutons from 40 randomly picked transformants. By performing another round transformation with the template DNA decreasing to 1 μg, 20 mutons were obtained, which was still less than the previous one without the template DNA. It indicated that excess foreign liner DNA may be toxic to the protoplast, but the AMA1 element will stabilize the foreign DNA maintained in the cell to alleviate the detriment. And it also proved our deduction that C. militaris contains a powerful SDSA‐HR repairing pathway to immediately fix the DSB caused by Cas9‐gRNA. Nevertheless, this section implied that the Cas9‐TRAMA system is capable of efficiently editing multiple sites and reconstructing genes in the genome via the NHEJ or HDR pathway of *C. militaris*.

### Application of the Cas9‐TRAMA system to protein modification, promoter strength evaluation and 10 kb cluster truncation in *C. militaris*

The Cas9‐TRAMA system was proved to have the ability of efficient editing the genome in *C. militaris*, and it has constructed a sequence truncated mutation of cordycepin synthetase Cns1 (Xia et al., 2017), which could be used for the function verification of its C‐terminal domain. To further test the genomic editing ability of the Cas9‐TRAMA system, we designed the other 3 pairs of gRNA targets to evaluate their function for native protein enrichment, promoter strength evaluation and 10 kb cluster deletion.

**FIGURE 4:**
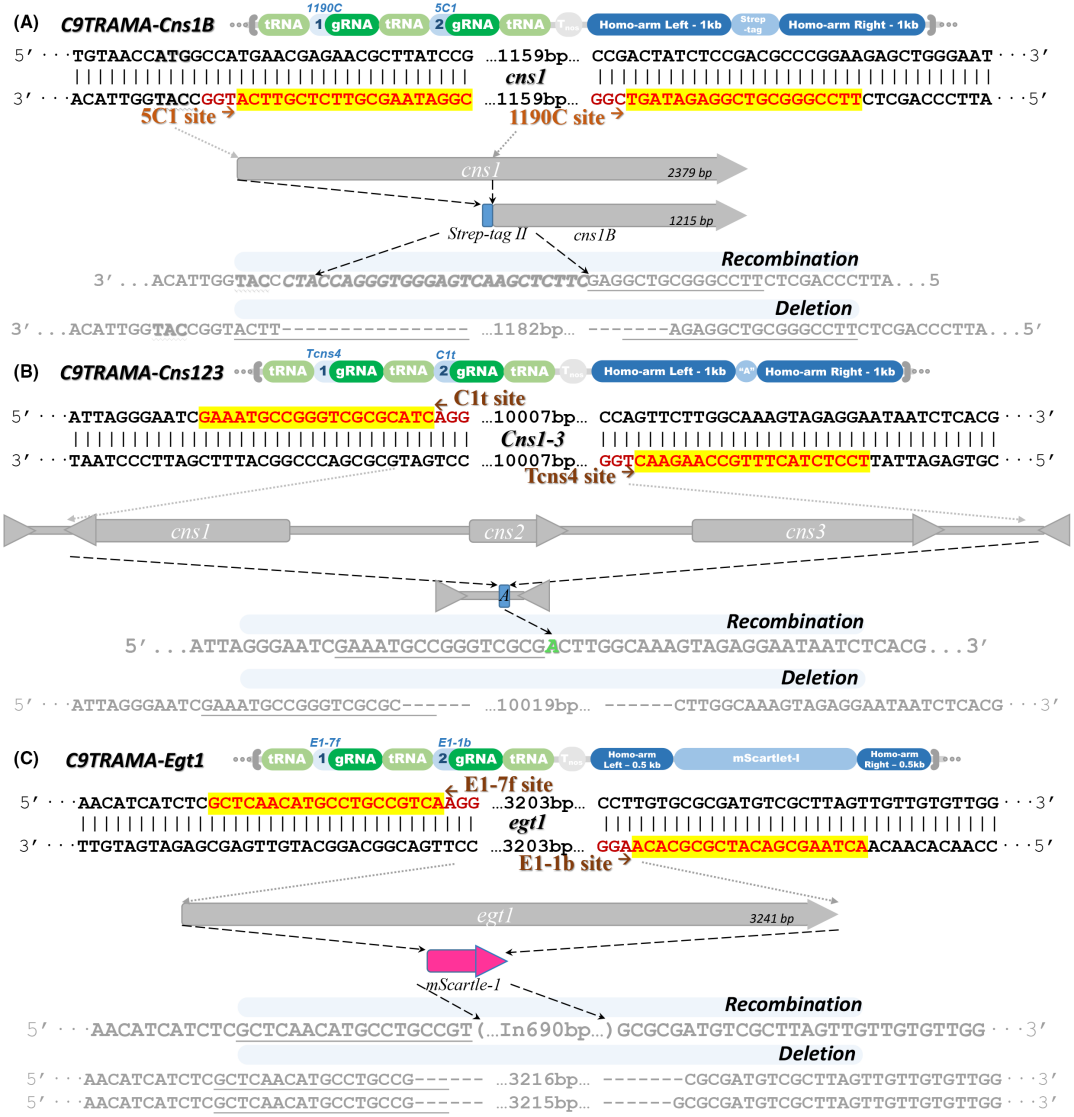
Diagram of the constitution of vector C9TRAMA‐Cns1B (A), ‐Cns123 (B) and ‐Egt1 (C). Alongside their designed of homologous templates and the sequencing result of mutons.

Previous research reported that the *cns1* was hard to express in *E. coli*, but the C‐terminal domain was structurally similar to the known function proteins, and it theoretically was the main function part of the Cns1. Fermentation of the previous Cns1B truncated muton showed that the absence of C‐terminal would lose its ability to synthesize cordycepin (data do not show in this study). So, in this section, we tried to further construct a muton with an N‐terminal domain replacing a strep‐tag peptide by the Cas9‐TRAMA system. This inserted peptide was fused to C‐terminal of *cns1* and endowed Cns1B capability to be enriched by Strep‐Tactin during the affinity purification. The locations of gRNAs were designed as shown in Figure 4A, only the target sites were edited and recombined as a template, the muton with strep‐ tag binding to Cns1B was constructed and the recombined Cns1B was controlled by the same promoter as the wild‐type strain. After transforming 2 μg of the C9TRAMA‐Cns1A vector into *C. militaris* and selecting by glufosinate ammonium plate, 10 mutons with double site editing were obtained by mycelium‐direct PCR based on different product lengths between wild‐type and mutons. Among them, 5 showed recombination as template DNA, while others showed truncation between 5 bp upstream of the PAM site in 5C1 and 1190C (Figure 4A, Table S5). After culture in an antibiotic‐free medium for one plate growth cycle, all mutons showed vector absence. They would further be used for cordycepin production verification and protein enrichment. To demonstrate the target protein was successfully labelled, a small‐scale shake‐flask cultivation of edited strain with strep‐tagged Cns1B was performed. As Figure S2 showed, after purification with resin for binding strep‐tagged protein and detection by protein electrophoresis, an enriched protein which size was consisted with the predicted size of Strep‐Cns1B was obtained. It indicated the Cas9‐TRAMA system can help to achieve native protein modification and purification in edible mushroom *C. militaris*.

Since most of the mushroom contain a lot of medicinal metabolites that were synthesized by unknown biosynthetic gene clusters, we next tested the large DNA fragment deletion function of the Cas9‐TRAMA system. Taking the cordycepin synthetic cluster as a target, a 10 kb length cluster contains the coding gene of *cns1*‐*3* and their promoters were selected as Figure 4B shows. To distinguish recombination and truncation, the template was assembled with 1 kb homologous arms and one single base insertion to form a restriction site. After the transformation with C9TRAMA‐Cns123, we obtained 16 mutons that showed recombination and 18 mutons that showed truncation. All truncations occurred between 3 bp upstream of the PAM sites in C1t and Tcns4. After removing the vector, the mutons were selected to perform fermentation and they all showed deficiency in producing its main metabolite cordycepin (Figure S3), while the wild type could produce cordycepin up to 2–4 g/L. This result indicated the efficient large cluster deleted function of the Cas9‐TRAMA system.

In addition, *C. militaris* has the tendency to develop as a chassis cell, but the molecular elements of the native cell were too limited to support metabolic engineering now. We sought to demonstrate that the Cas9‐TRAMA system can be used to evaluate promoter strength. The CCM_07351 gene was predicted to play a role in the synthesizing of ergothioneine in *C. militaris*. However, our previous transcriptome data (Chen et al., 2020) showed that this gene was barely transcribed during fermentation. We speculated that the promoter of CCM_07351 was under control by transcript factors or specific culture conditions, which means it could be developed as a potential molecular element for *C. militaris*. We therefore designed the gRNA located at the beginning and ending of the gene of CCM_07351. The template was designed to contain a fluorescent protein mscarlat‐1 (Bindels et al., 2017), around 500‐bp template arms on both sides (Figure 4C). The full length (3241 bp) of CCM_07351 was replaced with the coding gene (693 bp) of the fluorescent protein mScarlet‐1. With the construction of C9TRAMA‐Egt1 and the transformation, we obtained 2 recombined and 2 truncated mutons. The truncation occurred located between E1‐f7 and E1‐b1 sites. This result showed that shorter homologous arms would decrease the editing efficiency. But it also implied the Cas9‐TRAMA system is capable of coding gene replacement and further evaluating the metabolism‐regulating ability of local promoters.

All of these editing showed the wider application of the Cas9‐TRAMA system in *C. militaris*. Unfortunately, the off‐target problem was not a neglectable factor while applicated the CRISPR‐Cas9 system, and we indeed occasionally found fewer edited mutons showed degeneration, including slow growth and albino, in this study. However, mushroom is facing a serious problem of spontaneous degeneration. The normal strains may randomly generate mutation in a regular subculture. It is hard to estimate the certain reason for the degeneration now (Lou et al., 2019). We could not evaluate the off‐target effect of the Cas9‐TRAMA system in the current complex degeneration background of *C. militaris*. Nevertheless, the continuous expression of Cas9 is normally regarded as the reason for inducing off‐target effects. For alleviating the potential off‐target effect, the Cas9‐TRAMA system was designed to generate a sufficient amount of functional gRNA fragments and homology templates to constrain Cas9 to perform precise recognition during the genome editing stage. And once the strain was verified to be edited, the muton was sub‐cultured to non‐selective media so that the C9TRAMA vector could be spontaneously eliminated.

Besides, the construction of the C9TRAMA vector might be an obstacle to apply our Cas9‐TRAMA system in multiplex target editing. Because the original AMA1 fragment contains two 2.4‐kb length palindrome complementary sequences and many frequently used restriction sites, the construction of AMA1‐based vector is challenging. Though we applied the AMA1‐2.8 fragment, which is a shorter version of AMA1, in our Cas9‐TRAMA system to avoid the long terminal repeat problem, the multiple assembly of tRNA‐gRNA‐tRNA structure in the vector may also be a problem. To expand the application of the Cas9‐TRAMA system, we offered a detailed construction pathway and the key sequences of all vectors used in this study in Tables S1 and S2. Also, a schematic diagram of a methodology for efficiently replacing the target spacer sequence and adding extra sgRNA‐tRNA modules in the C9TRAMA vector was shown in Figure S4. With these detailed construction methods, we hope the diversified application of this efficient multiplex editing Cas9‐TRAMA technology will portend the rapid development of this mushroom in the future.

## CONCLUSIONS

In this study, we discovered the endogenic tRNA^Gly^ modified element in *C. militaris* and assemble it together with gRNA scaffold in the Cas9 vector to build a one‐step multiple target editing system using the ATMT method and selective pressure. With introducing the AMA1‐2.8 element and homologous template, the editing system was upgraded to a Cas9‐TRAMA genomic editing system, which can be efficiently edited multiple targets at any position of *C. militaris* genome. And more importantly, the Cas9‐TRAMA system could be removed without any unexpected foreign DNA leftover. We subsequently applied the Cas9‐TRAMA system in target sequence replacement (protein modification), foreign fluorescent protein coding gene precise positioning (promoter strength evaluation) and large fragment (10 kb biosynthetic cluster) deletion, which sought to demonstrate the benefit and wider applicated function in the genomic study of the Cas9‐TRAMA system. The Cas9‐TRAMA editing system provides a scalable method to excavate the valuable metabolic resource of edible mushroom and to engineer a mystical cellular pathway to elucidate the complex cell behaviours of mushroom‐forming fungi.

## AUTHOR CONTRIBUTIONS

CBX designed the study, performed major experiments, analysed the data and was a major contributor to writing the manuscript. XLN performed vector construction and transformation in *E. coli*. WT and YZW helped writing—review and editing the manuscript. WN and ZJR performed sequencing data curation. GLQ and LJF supervised the study, acquired funding and revised the paper. All authors read and approved the final manuscript.

## FUNDING INFORMATION

This work was supported by the China Postdoctoral Science Foundation (grant number 2020M682731); the National Natural Science Foundation of China (grant number 32072646, 31772373, 31801918). All data generated or analysed during this study are included in this publish article [and its supplementary information files].

## CONFLICT OF INTEREST

The authors declare that they have no competing interests.

## Supporting information


Supplementary Materials

## Data Availability

All data generated or analysed during this study are included in this publish article [and its supplementary information files].

## References

[mbt214147-bib-0001] Bindels, D.S. , Haarbosch, L. , van Weeren, L. , Postma, M. , Wiese, K.E. , Mastop, M. et al. (2017) mScarlet: a bright monomeric red fluorescent protein for cellular imaging. Nature Methods, 14, 53–56. 27869816 10.1038/nmeth.4074

[mbt214147-bib-0002] Boontawon, T. , Nakazawa, T. , Horii, M. , Tsuzuki, M. , Kawauchi, M. , Sakamoto, M. et al. (2021) Functional analyses of *Pleurotus ostreatus* pcc1 and clp1 using CRISPR/Cas9. Fungal Genetics and Biology, 154, 103599. 34153439 10.1016/j.fgb.2021.103599

[mbt214147-bib-0003] Boontawon, T. , Nakazawa, T. , Inoue, C. , Osakabe, K. , Kawauchi, M. , Sakamoto, M. et al. (2021) Efficient genome editing with CRISPR/Cas9 in *Pleurotus ostreatus* . AMB Express, 11, 30. 33609205 10.1186/s13568-021-01193-w PMC7897337

[mbt214147-bib-0004] Boontawon, T. , Takehito, N. , Xu, H. , Kawauchi, M. , Sakamoto, M. & Honda, Y. (2021) Gene targeting using pre‐assembled Cas9 ribonucleoprotein and split‐marker recombination in *Pleurotus ostreatus* . FEMS Microbiology Letters, 368, fnab080. 34156066 10.1093/femsle/fnab080

[mbt214147-bib-0005] Canino, G. , Bocian, E. , Barbezier, N. , Echeverría, M. , Forner, J. , Binder, S. et al. (2009) Arabidopsis encodes four tRNase Z enzymes. Plant Physiology, 150, 1494–1502. 19411372 10.1104/pp.109.137950 PMC2705019

[mbt214147-bib-0006] Čermák, T. , Curtin, S.J. , Gil‐Humanes, J. , Čegan, R. , Kono, T.J.Y. , Konečná, E. et al. (2017) A multipurpose toolkit to enable advanced genome engineering in plants. The Plant Cell, 29, 1196–1217. 28522548 10.1105/tpc.16.00922 PMC5502448

[mbt214147-bib-0007] Chan, P.P. , Lin, B.Y. , Mak, A.J. & Lowe, T.M. (2021) tRNAscan‐SE 2.0: improved detection and functional classification of transfer RNA genes. Nucleic Acids Research, 49, 9077–9096. 34417604 10.1093/nar/gkab688 PMC8450103

[mbt214147-bib-0008] Chen, B.‐X. , Wei, T. , Xue, L.‐N. , Zheng, Q.‐W. , Ye, Z.‐W. , Zou, Y. et al. (2020) Transcriptome analysis reveals the flexibility of cordycepin network in cordyceps militaris activated by L‐alanine addition. Frontiers in Microbiology, 11, 577. 32390960 10.3389/fmicb.2020.00577 PMC7193312

[mbt214147-bib-0009] Chen, B.‐X. , Wei, T. , Ye, Z.‐W. , Yun, F. , Kang, L.‐Z. , Tang, H.‐B. et al. (2018) Efficient CRISPR‐Cas9 gene disruption system in edible‐medicinal mushroom *Cordyceps militaris* . Frontiers in Microbiology, 9, 1157. 29946301 10.3389/fmicb.2018.01157 PMC6005869

[mbt214147-bib-0010] Chen, Y. , Wu, Y. , Li, S. , Du, S. , Hao, X. , Zhang, J. et al. (2021) Large‐scale isolation and antitumor mechanism evaluation of compounds from the traditional Chinese medicine *Cordyceps militaris* . European Journal of Medicinal Chemistry, 212, 113142. 33450619 10.1016/j.ejmech.2020.113142

[mbt214147-bib-0011] Cui, J.D. (2015) Biotechnological production and applications of *Cordyceps militaris*, a valued traditional Chinese medicine. Critical Reviews in Biotechnology, 35, 475–484. 24666119 10.3109/07388551.2014.900604

[mbt214147-bib-0012] Gutmann, B. , Gobert, A. & Giege, P. (2012) PRORP proteins support RNase P activity in both organelles and the nucleus in Arabidopsis. Genes & Development, 26, 1022–1027. 22549728 10.1101/gad.189514.112 PMC3360558

[mbt214147-bib-0013] Jan Vonk, P. , Escobar, N. , Wösten, H.A.B. , Lugones, L.G. & Ohm, R.A. (2019) High‐throughput targeted gene deletion in the model mushroom Schizophyllum commune using pre‐assembled Cas9 ribonucleoproteins. Scientific Reports, 9, 7632. 31113995 10.1038/s41598-019-44133-2 PMC6529522

[mbt214147-bib-0014] Katoh, K. & Standley, D.M. (2013) MAFFT multiple sequence alignment software version 7: improvements in performance and usability. Molecular Biology and Evolution, 30, 772–780. 23329690 10.1093/molbev/mst010 PMC3603318

[mbt214147-bib-0015] Liu, K. , Sun, B. , You, H. , Tu, J.‐L. , Yu, X. , Zhao, P. et al. (2020) Dual sgRNA‐directed gene deletion in basidiomycete *Ganoderma lucidum* using the CRISPR/Cas9 system. Microbial Biotechnology, 13, 386–396. 31958883 10.1111/1751-7915.13534 PMC7017817

[mbt214147-bib-0016] Lou, H. , Lin, J. , Guo, L. , Wang, X. , Tian, S. , Liu, C. et al. (2019) Advances in research on *Cordyceps militaris* degeneration. Applied Microbiology and Biotechnology, 103, 7835–7841. 31410524 10.1007/s00253-019-10074-z

[mbt214147-bib-0017] Lou, H. , Ye, Z. , Yun, F. , Lin, J. , Guo, L. , Chen, B. et al. (2018) Targeted gene deletion in *Cordyceps militaris* using the split‐marker approach. Molecular Biotechnology, 60, 380–385. 29605840 10.1007/s12033-018-0080-9

[mbt214147-bib-0018] Moon, S. , An, J.Y. , Choi, Y.‐J. , Oh, Y.‐L. , Ro, H.‐S. & Ryu, H. (2021) Construction of a CRISPR/Cas9‐mediated genome editing system in Lentinula edodes. Mycobiology, 49, 599–603. 35035251 10.1080/12298093.2021.2006401 PMC8725921

[mbt214147-bib-0019] Ouedraogo, J.‐P. & Tsang, A. (2020) CRISPR_Cas systems for fungal research. Fungal Biology Reviews, 34, 189–201.

[mbt214147-bib-0020] Qin, H. , Xiao, H. , Zou, G. , Zhou, Z. & Zhong, J.‐J. (2017) CRISPR‐Cas9 assisted gene disruption in the higher fungus Ganoderma species. Process Biochemistry, 56, 57–61.

[mbt214147-bib-0021] Reuter, J.S. & Mathews, D.H. (2010) RNAstructure: software for RNA secondary structure prediction and analysis. BMC Bioinformatics, 11, 129. 20230624 10.1186/1471-2105-11-129 PMC2984261

[mbt214147-bib-0022] Sarkari, P. , Marx, H. , Blumhoff, M.L. , Mattanovich, D. , Sauer, M. & Steiger, M.G. (2017) An efficient tool for metabolic pathway construction and gene integration for *Aspergillus niger* . Bioresource Technology, 245, 1327–1333. 28533066 10.1016/j.biortech.2017.05.004

[mbt214147-bib-0023] Sugano, S.S. , Suzuki, H. , Shimokita, E. , Chiba, H. , Noji, S. , Osakabe, Y. et al. (2017) Genome editing in the mushroom‐forming basidiomycete *Coprinopsis cinerea*, optimized by a high‐throughput transformation system. Scientific Reports, 7, 1260. 28455526 10.1038/s41598-017-00883-5 PMC5430836

[mbt214147-bib-0024] Tang, X. , Ren, Q. , Yang, L. , Bao, Y. , Zhong, Z. , He, Y. et al. (2019) Single transcript unit CRISPR 2.0 systems for robust Cas9 and Cas12a mediated plant genome editing. Plant Biotechnology Journal, 17, 1431–1445. 30582653 10.1111/pbi.13068 PMC6576101

[mbt214147-bib-0025] Tu, J.‐L. , Bai, X.‐Y. , Xu, Y.‐L. , Li, N. & Xu, J.‐W. (2021) Targeted gene insertion and replacement in the basidiomycete *Ganoderma lucidum* by inactivation of nonhomologous end joining using CRISPR/Cas9. Applied and Environmental Microbiology, 87, e0151021. 34524900 10.1128/AEM.01510-21 PMC8579997

[mbt214147-bib-0026] Waltz, E. (2016) Gene‐edited CRISPR mushroom escapes US regulation. Nature, 532, 293. 27111611 10.1038/nature.2016.19754

[mbt214147-bib-0027] Wang, P.‐A. , Xiao, H. & Zhong, J.‐J. (2020) CRISPR‐Cas9 assisted functional gene editing in the mushroom *Ganoderma lucidum* . Applied Microbiology and Biotechnology, 104, 1661–1671. 31865439 10.1007/s00253-019-10298-z

[mbt214147-bib-0028] Wang, T. , Yue, S. , Jin, Y. , Wei, H. & Lu, L. (2021) Advances allowing feasible pyrG gene editing by a CRISPR‐Cas9 system for the edible mushroom *Pleurotus eryngii* . Fungal Genetics and Biology, 147, 103509. 33400990 10.1016/j.fgb.2020.103509

[mbt214147-bib-0029] Wesseler, J. , Kleter, G. , Meulenbroek, M. & Purnhagen, K.P. (2022) EU regulation of genetically modified microorganisms in light of new policy developments: possible implications for EU bioeconomy investments. Applied Economic Perspectives and Policy, 44, 1–21.

[mbt214147-bib-0030] Xia, Y. , Luo, F. , Shang, Y. , Chen, P. , Lu, Y. & Wang, C. (2017) Fungal cordycepin biosynthesis is coupled with the production of the safeguard molecule pentostatin. Cell Chemical Biology, 24, 1–11. 29056419 10.1016/j.chembiol.2017.09.001

[mbt214147-bib-0031] Xie, K. , Minkenberg, B. & Yang, Y. (2015) Boosting CRISPR/Cas9 multiplex editing capability with the endogenous tRNA‐processing system. Proceedings of the National Academy of Sciences of the United States of America, 112, 3570–3575. 25733849 10.1073/pnas.1420294112 PMC4371917

[mbt214147-bib-0032] Zheng, P. , Xia, Y. , Xiao, G. , Xiong, C. , Hu, X. , Zhang, S. et al. (2011) Genome sequence of the insect pathogenic fungus *Cordyceps militaris*, a valued traditional chinese medicine. Genome Biology, 12, R116. 22112802 10.1186/gb-2011-12-11-r116 PMC3334602

[mbt214147-bib-0033] Zou, G. , Li, B. , Wang, Y. , Yin, X. , Gong, M. , Shang, J. et al. (2021) Efficient conversion of spent mushroom substrate into a high value‐added anticancer drug pentostatin with engineered *Cordyceps militaris* . Green Chemistry, 23, 10030–10038.

[mbt214147-bib-0034] Zou, G. , Xiao, M. , Chai, S. , Zhu, Z. , Wang, Y. & Zhou, Z. (2021) Efficient genome editing in filamentous fungi via an improved CRISPR‐Cas9 ribonucleoprotein method facilitated by chemical reagents. Microbial Biotechnology, 14, 2343–2355. 32841542 10.1111/1751-7915.13652 PMC8601184

